# The ATP binding site of the chromatin remodeling homolog Lsh is required for nucleosome density and *de novo* DNA methylation at repeat sequences

**DOI:** 10.1093/nar/gku1371

**Published:** 2015-01-10

**Authors:** Jianke Ren, Victorino Briones, Samantha Barbour, Weishi Yu, Yixing Han, Minoru Terashima, Kathrin Muegge

**Affiliations:** 1 Mouse Cancer Genetics Program, Center for Cancer Research, National Cancer Institute, Frederick, MD 21702, USA; 2 Basic Science Program, Leidos Biomedical Research, Inc., Mouse Cancer Genetics Program, Frederick National Laboratory for Cancer Research, Frederick, MD 21702, USA

## Abstract

Lsh, a chromatin remodeling protein of the SNF2 family, is critical for normal heterochromatin structure. In particular, DNA methylation at repeat elements, a hallmark of heterochromatin, is greatly reduced in Lsh^−/−^ (KO) cells. Here, we examined the presumed nucleosome remodeling activity of Lsh on chromatin in the context of DNA methylation. We found that dynamic CG methylation was dependent on Lsh in embryonic stem cells. Moreover, we demonstrate that ATP function is critical for *de novo* methylation at repeat sequences. The ATP binding site of Lsh is in part required to promote stable association of the DNA methyltransferase 3b with the repeat locus. By performing nucleosome occupancy assays, we found distinct nucleosome occupancy in KO ES cells compared to WT ES cells after differentiation. Nucleosome density was restored to wild-type level by re-expressing wild-type Lsh but not the ATP mutant in KO ES cells. Our results suggest that ATP-dependent nucleosome remodeling is the primary molecular function of Lsh, which may promote *de novo* methylation in differentiating ES cells.

## INTRODUCTION

DNA is packaged in chromatin which imposes a barrier for the interaction of proteins with DNA. Different molecular mechanisms can alter chromatin structure and control the accessibility of DNA binding factors. A number of DNA-based processes are regulated by chromatin accessibility including transcription, DNA repair and recombination (1,2).

Chromatin remodeling complexes alter chromatin structure in an adenosine triphosphate (ATP)-dependent manner. They target the nucleosome that is the smallest subunit of chromatin. Chromatin remodeling complexes are multi-subunit complexes, comprising of at least one SNF2 homolog and other accessory factors. SNF2 homologs belong to the SF2 family of helicases and contain more than 12 conserved sequence motifs (1,3). The SNF2 protein, comprising the catalytic subunit, performs nucleosome remodeling, and other members of the protein complex may reinforce this function (4). The ATP binding domain is highly conserved among SNF2 proteins and ATP hydrolysis is essential for nucleosome remodeling function (5,6). Several SNF2 family members are known as DNA translocases that utilize energy obtained from ATP hydrolysis to translocate along DNA. This would disrupt the interaction of DNA and proteins, most of which are histones components. Several SNF2 factors have been shown to shift nucleosomes along the DNA *in vitro*. Some SNF2 factors promote the exchange of specific histone proteins or induce assembly or disassembly of nucleosomes. SNF2 family members play important biological functions, such as transcriptional control, DNA repair and genomic recombination. The homologs Lsh (Mus musculus) and DDM1 (Arabidopsis thaliana) form their own subclass within the SNF2 family and are known to regulate DNA methylation during development (7–9).

DNA methylation is an important epigenetic mark that is predominantly detected at cytosines in the CpG (CG) context in mammals and found at CG and non-CG sites in plants (10–12). DNA methylation is performed by a class of highly conserved DNA methyltransferases. Deletion of DNA methyltransferase 1 (Dnmt 1) or Dnmt 3b is embryonic lethal in mice indicating a critical role in development. Blastocysts exhibit the lowest level of CG methylation during embryogenesis (13). A wave of *de novo* methylation is induced at gastrulation and tissue-specific DNA methylation patterns evolve in each cell type. The *in vivo* wave of DNA methylation can be recapitulated *in vitro* upon differentiation of embryonic stem (ES) cells (derived from the inner cell mass of blastocysts) into specific lineages (14,15). Thus *de novo* methylation accompanies cellular differentiation and the generation of tissue-specific gene expression patterns.

The SNF2 homologs Lsh and DDM1 are important regulators of DNA methylation in mice and plants, respectively. Lsh^−/−^ cells and DDM1 mutants show dramatic perturbation of DNA methylation, including a severe reduction of cytosine methylation at repeat elements (16–18). Transcriptional silencing of endogenous retrotransposons is a major function of DNA methylation. Consequently, Lsh mutant mice and DDM1 mutants display de-repression of hypomethylated repeat elements. Deletion of Lsh in mice is perinatal lethal, and embryos from knockout mice have numerous organ and developmental defects (19–24). Knockdown of Lsh in ES cells by siRNA interference reduces DNA methylation at pluripotency genes (23). Although pluripotency genes show impaired repression in siLsh-treated ES cells upon *in vitro* differentiation, Lsh^−/−^ embryos effectively silence Oct4 and show cellular differentiation despite incomplete CG methylation indicating that cellular differentiation can take place in hypomethylated stem cells (23,25). The chromatin of Lsh^−/−^ fibroblasts displays not only reduced DNA methylation, but also alterations in histone modifications, such as changes in H3K4me3, H3K27me3 and H3K9me3 (16,18,25–29). However, histone modification changes are dispersed at specific genomic locations, whereas the decrease in CG methylation is widespread and comprises about 30% of all cytosines in the CG context (18). Likewise, the major epigenetic modifications that are detected in DDM1 mutants are alterations of DNA methylation. For this reason, it is thought that the primary role of Lsh/DDM1 is the regulation of DNA methylation patterns.

Based on the homology in SNF2 family, DDM1 and Lsh have presumed chromatin remodeling activity, but this activity has only been demonstrated for DDM1 (30). Recent genome wide bisulfite sequencing analysis revealed substantial reduction of cytosine methylation at histone 1 (H1) rich regions in DDM1 mutants (17). In fact, DDM1 is only required for the generation of DNA methylation patterns, when H1 is present. This suggests that DDM1 promotes methylation at H1 (linker DNA) rich regions that are thought to be packaged into a more dense form of chromatin. Since nucleosome DNA imposes a barrier on DNA methylation *in vitro*, which can be alleviated by SNF2 factors, it has been hypothesized that DDM1 promotes access of DNA methyltransferases to nucleosome DNA at H1 dense repressed chromatin (31). Likewise, genome wide assessment of DNA methylation revealed that Lsh was important for DNA methylation at the nuclear compartment that was in part defined by lamin B1 attachment sites, a genomic region rich in repressed and dense chromatin (18).

Lsh controls DNA methylation levels in mice, but it remains uncertain how Lsh is involved in this process. In particular, it remains unclear whether Lsh is acting directly or indirectly to control DNA methylation, whether chromatin remodeling is performed and required or whether Lsh serves solely as a scaffolding protein to promote assembly of the DNA methylation pathway. In order to study the primary molecular function of Lsh on chromatin, we established a dynamic assay allowing us to monitor *de novo* methylation. We generated ES cells with a deletion of Lsh and re-constituted the cells with full-length Lsh or mutant Lsh proteins. We report here distinct functional properties of Lsh domains and provide evidences of chromatin remodeling function by Lsh *in vivo*.

## MATERIALS AND METHODS

### ES cell culture and differentiation

ES cells were cultured in Knockout™ D-MEM (Invitrogen) media supplemented with 15% Fetal Bovine Serum (FBS, Invitrogen), Minimum Essential Media (MEM) nonessential amino acids 1× (Invitrogen), GlutaMAX-1× (Invitrogen), 0.1-mM 2-mercaptoethanol (Sigma), penicillin-streptomycin (Invitrogen), mouse Leukemia Inhibitory Factor (LIF) (Millipore) plus PD0325901 (Stemgent), CHIR99021 (Stemgent) in 0.1% gelatin (Sigma) coated plates. Cells were passaged after dissociation with trypsin and plated every other day. ES cells were derived as previously described (32). All ES cell lines were derived from D3.5 embryos generated by crossing Lsh^+/−^ heterozygotes (33). To induce differentiation into the neuroepithelial lineage, ES cells were cultured in ES medium for 4 days without LIF for embryoid body (EB) formation, then treated with 1-μM retinoic acid (RA) (Sigma) for another 4 days, while the cell culture medium was changed every other day.

### Plasmids and electroporation of ES cell

Full-length wild-type Lsh cDNA, Lsh binding site mutant (K237A) or DEAH domain deletion cDNAs were subcloned in frame with an N-terminal 3× Flag tag driven by CAG promoter. About 10 million cells were electroporated with plasmid DNA in phosphate buffered saline (PBS) and then seeded on a 100-mm 0.1% gelatin-coated dish. Recombinant colonies were selected in hygromycin containing media (Gibco) for 7–10 days. Viable colonies were hand-picked and Lsh expression validated by real-time polymerase chain reaction (PCR), immunofluorescence staining and western blot analysis.

### Western blot

Cells were lysed in buffer containing 150-mM NaCl, 50-mM Tris-HCl, pH 7.5, 0.5% Triton X-100 (v/v) and protease-inhibitor cocktail (Roche). Homogenized protein (10 μg) was loaded onto a 10% acrylamide/bis gel and transferred to a Polyvinylidene difluoride (PVDF) membrane after electrophoresis. Following blocking with 5% nonfat milk for 1 h, membranes were incubated at 4°C overnight with primary antibodies: Anti-Flag, M2 (sigma, F1804); Lsh (polyclonal rabbit-antiserum raised against recombinant Lsh); Anti-Actin (Sigma, A2228). Horseradish peroxidase-conjugated secondary antibodies were incubated for 1 h at room temperature. Amersham Enhanced Chemiluminescence (ECL) western blotting analysis system was used for blot detection.

### Immunofluorescence staining

ES cells grown on chamber slides (ibidi, 80826) were briefly washed in PBS and fixed in 4% paraformaldehyde (Sigma, prepared in PBS) for 20–25 min at room temperature. The cells were subsequently permeabilized with 0.2% Triton X-100 in PBS (15 min), washed in PBS and blocked in 1% bovine serum albumin (BSA) (30 min). Cells were then incubated with the indicated antibody in the same buffer at 4°C overnight. The slides were subsequently washed three times in 1% BSA–PBS and incubated with Alexa fluorophore–conjugated secondary antibodies (Invitrogen) for 1 h at room temperature in the dark and washed three times. Finally, the slides were stained with 4',6-diamidino-2-phenylindole (DAPI) and imaged by confocal microscopy. These following antibodies were used: Anti-Flag, M2 (sigma, F1804); Lsh (polyclonal rabbit anti-Lsh); Oct4 (Stemgent, 09-0023); Nanog (Stemgent, 09-0020); Sox2 (Abcam, ab75485); SSEA-1 (Stemgent, 09-0067); Nestin (Abcam, ab11306); Tubb3 (Abcam, ab18207).

### qRT-PCR analysis

For qRT-PCR, total RNA was isolated from ES sample using Qiagen RNeasy Mini kit with DNase treatment and random-primed reverse transcription was performed by using the Invitrogen Superscript III kit on 1-μg RNA. The real-time quantitative PCR was performed by using SYBR Green dye on Bio-Rad MyiQ2 system. Relative expression was normalized to internal Gapdh abundance.

### ChIP-qPCR assays

ChIP (chromatin immunoprecipitation) assays were carried out as EZ Chip kit (Millipore) described (34). Briefly, cells were cross-linked with 1% formaldehyde, lysed and sonicated on ice to generate DNA fragments with an average length of 200–1000 bp. One percent of each sample was saved as input fraction. Immunoprecipitation was performed using specific antibodies against the indicated proteins or IgG as control. After reversal of cross-linking, DNA was prepared for qPCR analysis. The following antibodies were used: Lsh (polyclonal rabbit anti-Lsh); Dnmt3b (Imgenex, IMG-184A); rabbit/Mouse IgG (Millipore). Precipitated DNA was re-suspended in 50 μl of Nuclease-Free water (Invitrogen) and analyzed by qPCR using the specific primers shown in Supplementary Table S1.The normalization method for ChIP analysis is percent of input. Each ChIPs result represents three samples (mean and SD of *n* = 3). For *P*-value computation, the student *t*-test was applied.

### Bisulfite conversion assay

About 0.5-μg genomic DNA was subjected to bisulfite treatment using MethylDetector kit (Active Motif). The PCR products were separated in agarose gels, purified and subcloned as described (27). More than 10 clones for each sample were sequenced to assess the methylation status of cytosine. The methylation level was presented as CG methylation ratio (fraction of methylated cytosine over total cytosine).

### Nucleosome occupancy assay

Nucleosome occupancy assay was performed using NOMe-Seq kit (Active Motif) as described (35–37). Briefly, cells were trypsinized and centrifuged for 3 min at 500 × g, then washed in ice-cold PBS and resuspended in 1-ml ice-cold nuclei buffer (10-mM Tris [pH 7.4], 10-mM NaCl, 3-mM MgCl2, 0.1-mM ethylenediaminetetraacetic acid (EDTA) and 0.5% NP-40, plus protease inhibitors) per 5 × 10^6^ cells and incubated on ice for 10 min. Nuclei were recovered by centrifugation at 900 × g for 3 min and washed in nuclei wash buffer (10-mM Tris [pH 7.4], 10-mM NaCl, 3-mM MgCl2 and 0.1-mM EDTA containing protease inhibitors). Freshly prepared nuclei (2 × 10^5^ cells) were sonicated to generate fragments of more than 1 kb, then treated with 200 U of M.CviPI (NEB) in 15 μl 10× reaction buffer, 45 μl 1-M sucrose and 0.75-μl S-adenosyl methionine (SAM) in a volume of 150 μl. Reactions were quenched by the addition of an equal volume of Stop Solution (20-nM Tris-HCl [pH 7.9], 600-mM NaCl, 1% sodium dodecyl sulphate, 10-mM EDTA, 400-μg/ml Proteinase K) and incubated at 55°C overnight. The chromatin was subjected to reversal crosslink, RNAase A and Protein K treatment then purified by phenol/chloroform extraction and ethanol precipitation. Bisulfite conversion was performed using the MethylDetector kit (Active Motif). PCR products were separated and cloned using the TA Kit (Qiagen), both according to the manufacturers’ instructions.

## RESULTS

### Lsh regulates dynamic DNA methylation patterns at repeat sequences

In order to study the molecular function of Lsh on chromatin, we established an *in vitro* ES cell-based system. DNA methylation levels vary during development and are lowest in the inner cell mass of blastocysts before implantation (10). Tissue differentiation after implantation is associated with the establishment of *de novo* DNA methylation. ES cells are derived from the inner cell mass and show globally lower CG methylation level compared to somatic cells (18). In order to establish a dynamic assay and characterize roles for Lsh in chromatin remodeling and DNA methylation, we generated Lsh^−/−^ (KO) ES cells and Lsh^+/+^ (WT) ES cells by deriving them from day 3.5 blastocysts. The KO ES and WT ES cells exhibit characteristic pluripotency markers such as Oct4, Sox2, SSEA1 and Nanog, they also display similar growth rates *in vitro* (Supplementary Figures S1 and S2). Our previous analysis, using siLsh-treated ES cells and Lsh^−/−^ tissues, had indicated that Lsh was critical for complete CG methylation at the Oct4 gene and siLsh-treated ES cells showed a subpopulation with sustained Oct4 expression (23). However, Lsh^−/−^ tissues effectively silenced Oct4 and showed cellular differentiation despite reduced CG methylation indicating a redundancy of epigenetic silencing pathways *in vivo* (23). Despite a reduction in CG methylation at the Oct4 promoter regions (40% in KO ES cells versus 67% in WT cells, data not shown), KO ES cells were able to differentiate into the neuroepithelial lineage. The efficiency of cellular differentiation was comparable with WT ES cells, as judged by staining for neuronal epithelial markers Tubb3 and Nestin and examining mRNA expression of several neural lineage genes (Supplementary Figure S2). This indicated that cellular differentiation took place in KO ES cells at a comparable rate despite incomplete CG methylation.

**Figure 1.**
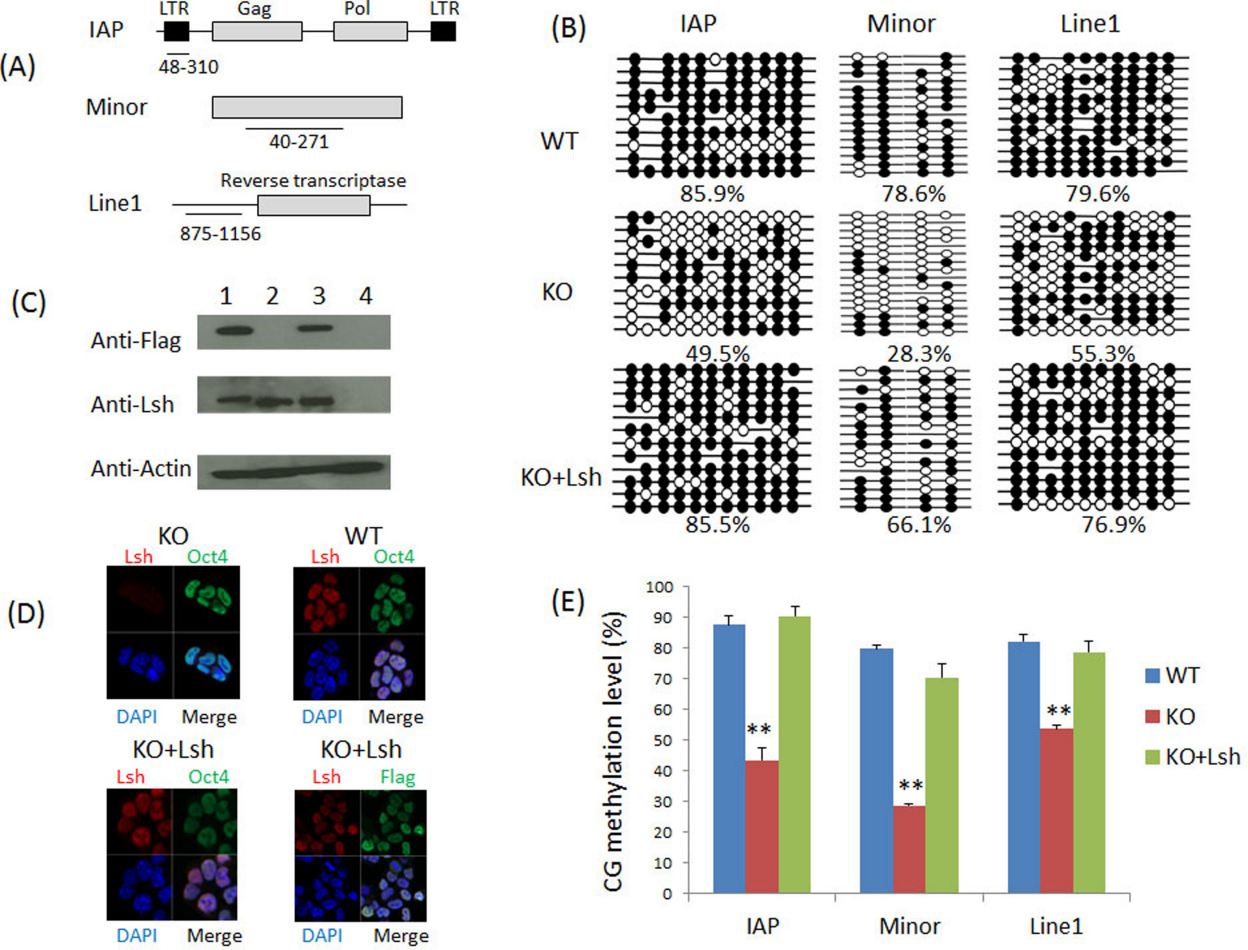
Re-induction of Lsh in KO ES cell restores DNA methylation during ES differentiation. (**A**) The regions analyzed by bisulfite sequencing in IAP LTR (5′ long terminal repeats), minor satellite sequence and Line1 elements are shown in a schematic graph. (**B**) Bisulfite sequencing analysis of the IAP elements, minor satellite sequences and Line1 elements in RA-treated WT ES cells (WT), KO ES cells (KO) and KO ES cells re-expressing full-length Lsh protein (KO+Lsh). A detailed RA differentiation protocol is shown in Supplementary Figure S2B. The black filled circles represent CG methylation, while the white open circles indicate no methylation at specific CpG sites. Gaps in the methylation profiles indicate mutated or missing CpG sites. The percentage of methylated CpGs is shown below each group of clones. Each datum represents one representative experiment of two to four independent experiments (see Figure 1E). (**C**) Western analysis for detection of flag-tagged Lsh protein. Undifferentiated KO ES cells transfected with Lsh expression vector (1), WT ES cells (2), WT ES cells re-expressing full-length wild-type Lsh (3) and KO ES cells (4). (**D**) Immune fluorescence analysis for detection of the nuclear location of Lsh in undifferentiated KO ES cells expressing full-length wild-type Lsh (KO+Lsh) using an anti-Lsh antibody or the antibody against the flag epitope. WT ES cells were used as positive control, KO ES cells served as negative control. Oct4 antibodies were used for detection of the pluripotency marker Oct4. Nuclei were stained with DAPI. (**E**) Bar graph representing CG methylation level in RA-treated WT ES cells (WT), KO ES cells (KO) and KO ES cells re-expressing full-length wild-type Lsh protein (KO+Lsh) at IAP sequences, minor satellite sequences and Line1 elements. The graph represents mean ± SD from four independent experiments (IAP, Line1) or two independent experiments (minor satellite), each including at least 10 sequenced clones. SD: standard deviation. *P < 0.05; **P < 0.01.

First, we examined CG methylation level at several repeat elements in WT and KO ES cells after ES differentiation using RA treatment (Figure 1A). About 44% of DNA sequences in the murine genome are derived from repeat sequences, and comprise minor satellite sequences (present at centromeric regions), major satellite sequences (present at pericentromeric regions), endogenous retroviral elements containing long terminal repeats (LTR) (such as the IAP, intracisternal A particle) and rudiments of retrotransposons known as LINE (long interspersed) elements (38). Repeat sequences are frequently hypomethylated in cancer and are thought to play an important role in genome stability. Using bisulfite sequencing we observed that after RA induced differentiation, WT ES cells showed higher CG methylation compared to KO ES cells at IAP sequences (86% versus 49%), minor satellite sequences (79% versus 28%) and Line1 elements (80% versus 55%) (Figure 1B). This indicates that the level of CG methylation at repeat sequences is reduced in the absence of Lsh. Interestingly, RA-induced differentiation was required to gain full DNA methylation competence in ES cells. In particular, minor satellite sequences and Line1 elements increased in WT CG methylation after differentiation from 59 to 79% and from 26 to 80%, respectively (Figure 1B and Supplementary Figure S3). In contrast, CG methylation level did not increase in KO ES cells after RA induced differentiation at minor satellite sequences and the acquisition of methylation was impaired at Line1 elements (Supplementary Figure S3).

To test whether the gain of DNA methylation depends directly on Lsh, we re-introduced full-length wild-type Lsh into KO ES. KO ES cells reconstituted with Lsh exhibit comparable Lsh protein level relative to WT ES cells (Figure 1C and D). Bisulfite sequencing revealed that Lsh restored CG methylation to wild-type levels at the IAP sequences and Line1 elements and close to wild-type levels at minor satellite sequences in RA-treated ES cells (Figure 1B and E). This indicates that the establishment of DNA methylation patterns at repeat sequences upon cellular differentiation depends on the presence of Lsh.

Remarkably, the restoration of CG methylation patterns upon re-introduction of Lsh in KO ES cells required RA-stimulated differentiation. Thus, undifferentiated cells displayed a similar CG methylation at IAP and minor satellite sequences with or without Lsh (42% versus 41% and 23% versus 25%, respectively) (Supplementary Figure S3). Likewise, complete CG methylation in WT ES cells was only achieved after RA induced differentiation (as mentioned above). It is presently unknown, what ‘component’ of the DNA methylation pathway is promoted by RA-induced differentiation (as discussed below), since Lsh and Dnmt3a/b proteins are already highly expressed in undifferentiated ES cells (23).

In summary, we describe here a dynamic assay of *de novo* methylation that depends on the presence of Lsh for complete DNA methylation.

### Functional domains of Lsh are required to promote DNA methylation at repeat sequences

**Figure 2.**
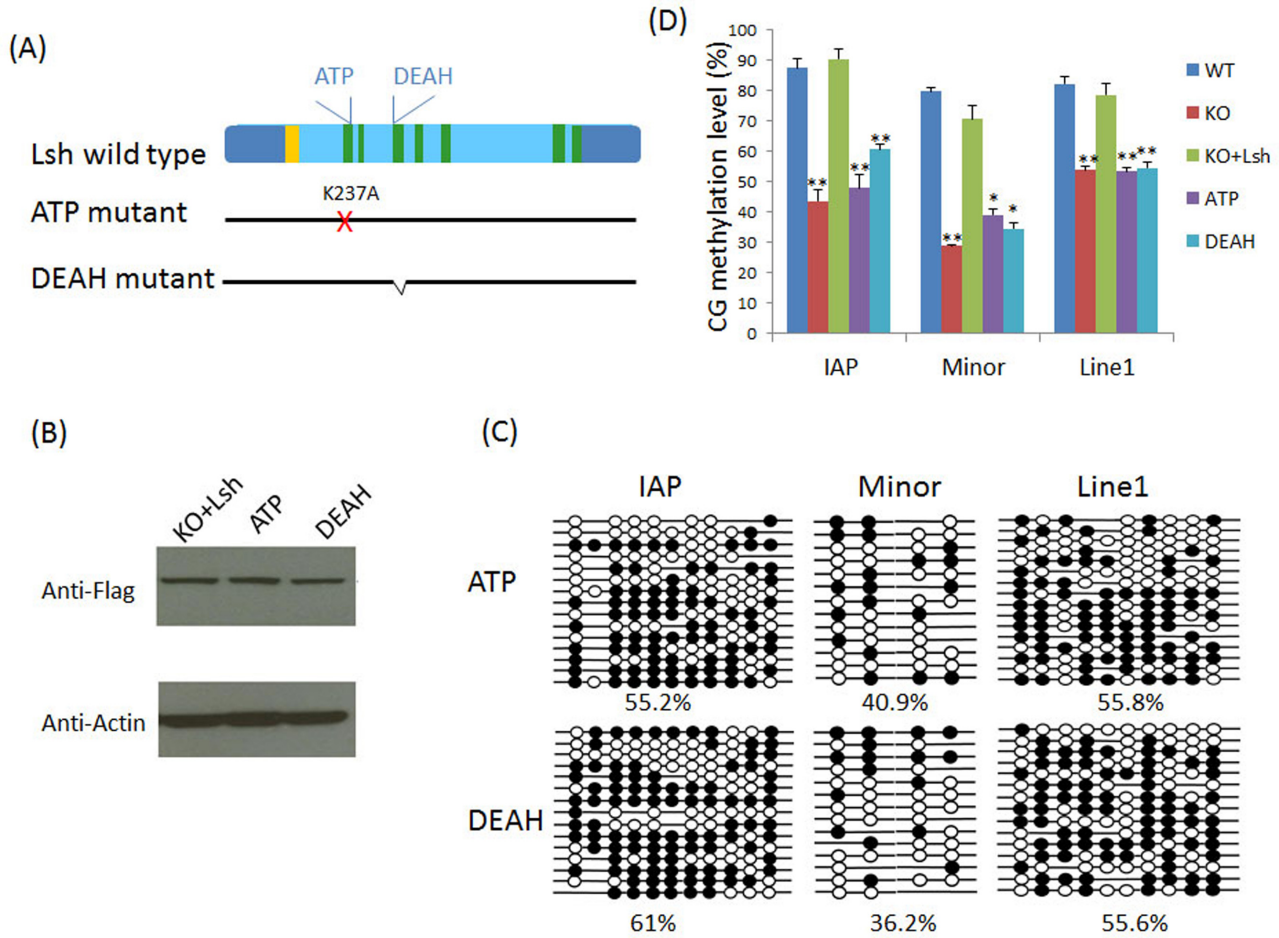
The ATP binding site is required for efficient CG methylation at repeat elements. (**A**) Schematic representation of the seven conserved domains (green) in full-length wild-type Lsh including the nuclear localization sequence (yellow). The ATP mutant contains a point mutation exchanging the lysine (K) at amino acid 237 to alanine (A). The deletion of the conserved DEAH box comprises amino acids 325 to 342. (**B**) Western analysis for detection of the Lsh ATP binding site mutant protein (ATP) and DEAH deletion protein (DEAH) compared to full-length wild-type Lsh protein (KO+Lsh) in undifferentiated KO ES cells. (**C**) Bisulfite sequencing analysis of the IAP elements, minor satellite sequences and Line1 elements in RA-treated KO ES cells re-expressing the ATP mutant protein (ATP) or the DEAH mutant protein (DEAH). The black filled circles represent CG methylation, while the white open circles indicate no methylation at specific CpG sites. The data represent one representative experiment of two to four independent experiments (see below). (**D**) Bar graphs showing relative CG methylation level in RA-treated KO ES cells re-expressing full-length wild-type Lsh (KO+Lsh), ATP mutant protein (ATP) or DEAH mutant protein (DEAH). For comparison RA-treated WT ES cells and KO ES cells are used. The data summarize four independent experiments for IAP and Line1 elements, with the exception of *n* = 3 for the DEAH mutant and *n* = 2 for minor satellites. Data present mean ± SD. SD: standard deviation. *P < 0.05; **P < 0.01.

In order to define functional domains of the Lsh protein, we generated Lsh expression vectors with a point mutation in the ATP binding site or a deletion of the DEAH box, a highly conserved motif in all SNF2 family members (Figure 2A) (8). The ATP binding site is required for putative chromatin remodeling activity. A Point mutation at the ATP binding site (converting a conserved lysine at position 237 to an arginine) in SNF2 factors abrogates their ability to hydrolyze ATP and therefore the SNF2 factor can no longer mobilize nucleosomes *in vitro* and *in vivo* (5,39,40).

Both mutant Lsh proteins were successfully expressed in KO ES cells and their protein abundance was similar to the level of full-length wild-type Lsh in KO ES cells, as demonstrated by western analysis (Figure 2B). Since wild-type Lsh rescues CG methylation only in differentiated ES cells, we performed bisulfite sequencing analysis after RA induced differentiation to compare the effect of ATP or DEAH mutation on DNA methylation. RA-treated ES cells harboring the ATP mutant Lsh protein displayed reduced CG methylation compared to full-length wild-type Lsh with CG methylation level ∼55% versus 86%, ∼41% versus 66% and 56% versus 77% at IAP, minor satellite and Line1 sequences, respectively (Figure 2C). Likewise, RA-treated KO cells restored with the DEAH box Lsh mutant protein exhibit reduced CG methylation compared to wild-type Lsh protein, as shown in Figure 2C, and these methylation levels are similar to those in KO ES (Figure 2D). Thus neither mutant Lsh protein was able to completely restore Lsh function compared to wild-type Lsh protein (Figure 2D). This indicates that both domains, the ATP binding site and the DEAH box, are required to facilitate the establishment of methylation patterns at those repeat sequences upon ES differentiation.

### The role of Lsh domains in facilitating Dnmt3b association

**Figure 3.**
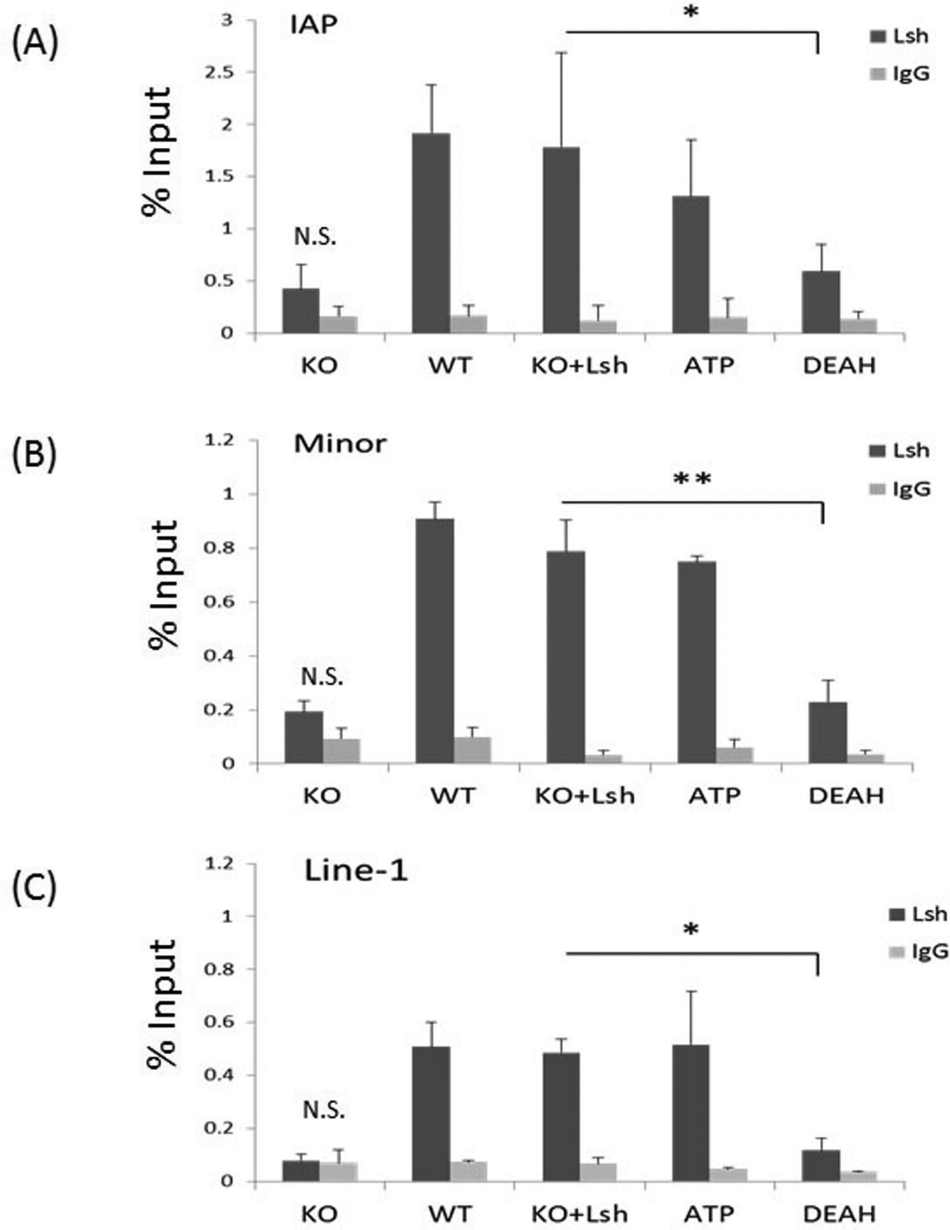
Association of mutant Lsh protein with repeat sequences. ChIPs analysis followed by real-time PCR analysis to assess Lsh enrichment at IAP sequences (**A**), minor satellite sequences (**B**) and Line1 elements (**C**) in undifferentiated ES cells of wild-type origin (WT), KO ES cells (KO) and KO ES cells re-expressing full-length wild-type Lsh (KO+Lsh), the ATP mutant (ATP) or the DEAH domain deletion (DEAH). The immunoprecipitation signal in KO ES cells is not significantly elevated (N.S.) above the IgG control. ChIPs results represent the mean ± SD of three independent experiments. SD: standard deviation. *P < 0.05; **P < 0.01.

Since we had previously observed that the presence of Lsh in ES cells promotes Dnmt3b association at genomic loci such as the Oct4 promoter region (23), we performed ChIPs analysis to determine the association of Lsh and Dnmt3b proteins with repeat sequences. As shown in Figure 3, Lsh is associated with repeat loci in undifferentiated cells. This suggests a local (direct) role of Lsh in the subsequent event of *de novo* methylation, as opposed to inducing a factor that in turn controls DNA methylation. The ATP Lsh mutant protein does not affect its enrichment at IAP, minor satellite sequences and Line1 elements compared to Lsh in undifferentiated WT ES cells (Figure 3). This indicates that the ATP binding site is dispensable for Lsh association with those genomic loci. In contrast, the DEAH box mutant Lsh protein displayed reduced binding at repeat sequences suggesting a critical role for this motif in Lsh binding to chromatin targets. However, we cannot exclude the possibility that the anti-Lsh antibody does not effectively recognize the mutant Lsh protein in the chromatin context. The limited sensitivity of our ChIP assay may in part explain that we observed a small increase in CG methylation (Figure 2D) despite the low enrichment of the DEAH box mutant protein above background (Figure 3).

**Figure 4.**
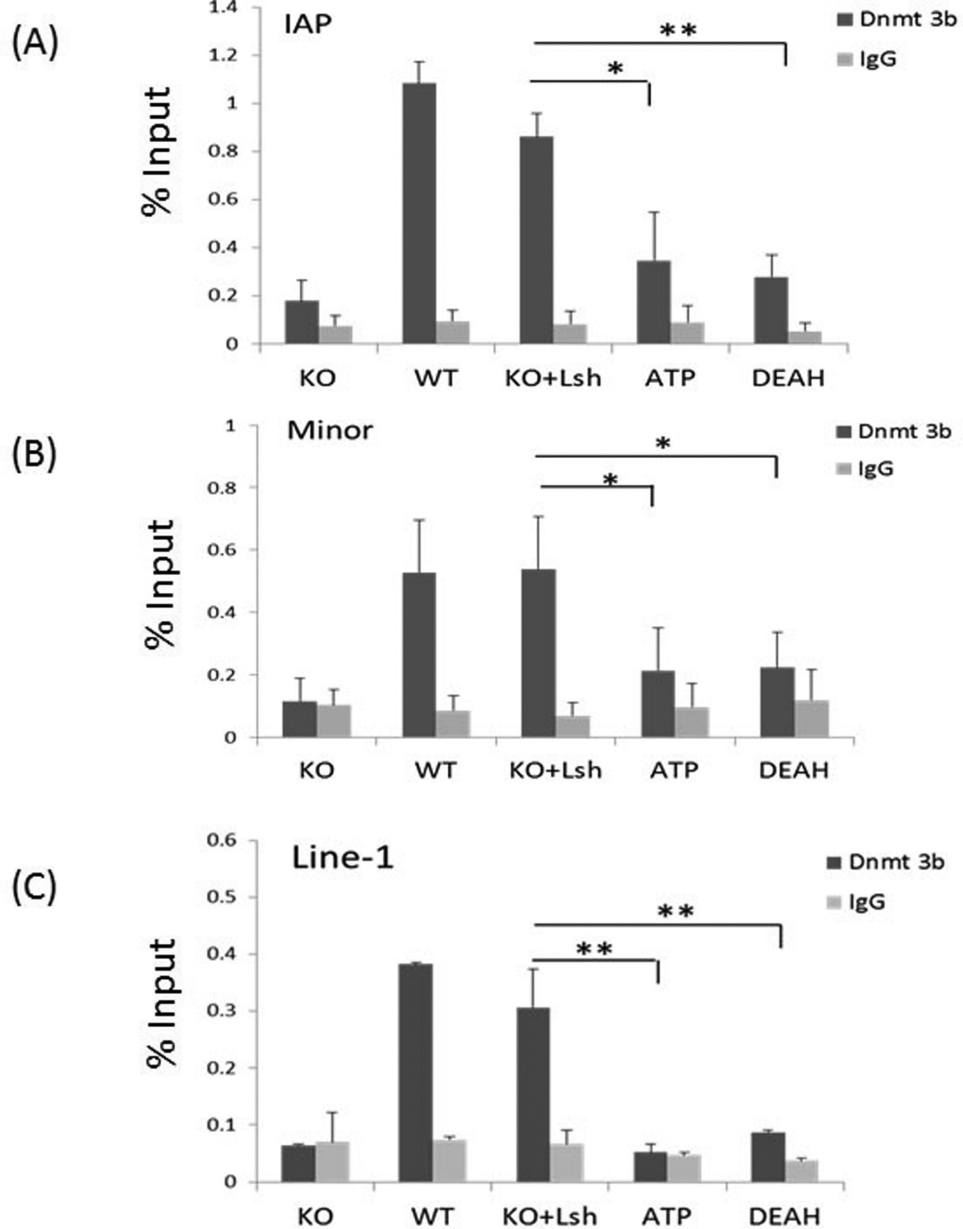
Association of Dnmt3b with repeat sequences in KO ES cells restored with mutant Lsh protein. ChIPs analysis followed by real-time PCR analysis to assess Dnmt3b enrichment at IAP sequences (**A**), minor satellite sequences (**B**) and Line1 elements (**C**) in undifferentiated ES cells of wild-type origin (WT), KO ES cells (KO) and KO ES cells re-expressing full-length wild-type Lsh (KO+Lsh), the ATP mutant (ATP) or the DEAH domain deletion (DEAH). ChIPs using IgG served as the controls. ChIPs results represent the mean ± SD of three independent experiments. SD: standard deviation. *P < 0.05; **P < 0.01.

Furthermore, we examined association of Dnmt3b with repeat sequences comparing undifferentiated WT and KO ES cells (Figure 4). We observed that binding of Dnmt3b to IAP, minor satellite sequences and Line1 elements loci is reduced in KO ES cells compared to WT ES cells (Figure 4). Moreover, Dnmt3b association is restored after re-expression of full-length wild-type Lsh in KO ES cells, indicating that Dnmt3b occupancy at those repeat sequences depends on the presence of Lsh. Next, we examined the ability of mutant Lsh protein to promote association of Dnmt3b with chromatin targets. We hypothesized that Lsh may either serve as scaffolding protein and may directly recruit Dnmts (28) or ATP function may be involved. As shown in Figure 4, undifferentiated KO ES cells that had been transfected with Lsh mutant proteins showed greatly impaired Dnmt3b binding compared to the effect of the full-length wild-type Lsh protein. This indicates critical roles of the ATP binding site and the DEAH motif of Lsh in recruiting Dnmt3b to repeat elements. Since the DEAH box mutant protein shows reduced association with repeat sequences, it is possible that either presumed chromatin remodeling function or a scaffolding function of Lsh is responsible for changes in Dnmt3b binding. However, the failure of the ATP mutant protein to restore Dnmt3b association implies an important role for ATP function of the Lsh protein.

Taken together, Lsh is required to promote a gain of DNA methylation at repeat elements upon ES cell differentiation. The ATP function of Lsh is critical for *de novo* methylation at repeat sequences. In addition, The ATP function plays, in part, a role in promoting association of Dnmt3b with repeat sequences.

### Nucleosome occupancy depends on the ATP function of Lsh

In order to explore the primary function of Lsh and to understand the molecular mechanism of the ATP binding site, we examined nucleosome occupancy at repeat elements in KO and WT using the Nucleosome Occupancy and Methylome (NOMe) sequencing assay. This method is a high-resolution single molecule assay that provides both nucleosome footprint and a DNA methylation profile at the same time (35–37). Bacterial GpC methyltransferase (M.CviPI) is used to treat fixed chromatin to artificially methylate GpC dinucleotides that are not protected by nucleosomes or other proteins bound to the DNA. Although the bacterial enzyme does not successfully methylate all available GpC sites, possibly due to experimental limitations, the effective methylation of GpC sites provides evidence of accessibility of the M.CviPI enzyme to these regions that preclude nucleosome occupancy. Treated DNA is subjected to bisulfite conversion and gene-specific loci are sequenced. By combining the sequencing information for the GpC sites (artificially introduced) with the methylation at CpG sites (*in vivo* methylation patterns), a profile of nucleosome occupancy (≥146 bp) and DNA methylation for a specific gene locus can be determined. Recent genome wide analysis has validated this technique and revealed access of M.CviPI to linker DNA at well-positioned nucleosomes flanking CTCF binding sites and confirmed nucleosome-depleted regions upstream of transcriptional start sites (36).

**Figure 5.**
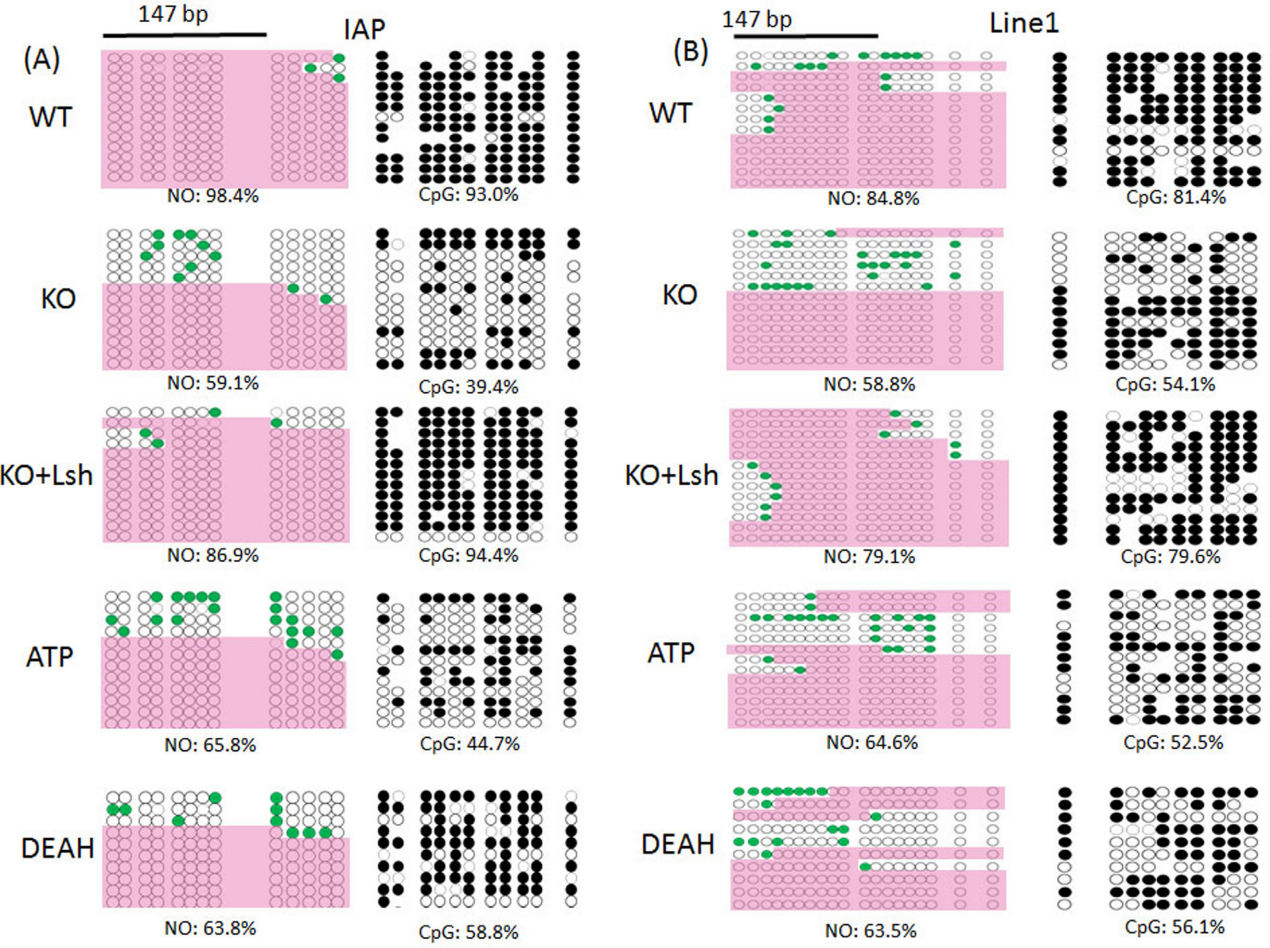
Nucleosome occupancy assay at repeat sequences in KO ES cells restored with mutant Lsh protein. NOMe assay of IAP 5′LTR (**A**) and Line1 5′region (**B**) in RA-treated ES cells of wild-type origin (WT), KO ES cells (KO) and KO ES cells re-expressing full-length wild-type Lsh (KO+Lsh), the ATP mutant (ATP) or the DEAH domain deletion (DEAH). The GpC methylation profile is shown on the left-hand and represents GpC sites that are not methylated (white circle) or methylated (green-filled circles) indicating the accessibility of those GpC sites to GpC methyltransferase. The areas of inaccessibility, large enough to accommodate a nucleosome (≥147 bp), are identified as described (35–37) and are covered by pink color. The CpG methylation profiles are shown on the right with methylated (black circle) and unmethylated (white circles) cytosines. NO: nucleosome occupancy. CpG: CpG methylation. The data represent one out of *n* = 3 (WT, KO, KO+Lsh, ATP) or *n* = 2 (DEAH) independent experiments.

First, we applied the NOMe assay to evaluate nucleosome occupancy in undifferentiated WT and KO ES cells at IAP sequences and Line1 elements, since minor satellites sequences do not contain sufficient numbers of GpC sites to perform this assay. Undifferentiated WT and KO ES cells displayed high nucleosome occupancy (Supplementary Figure S4). Since undifferentiated KO ES cell displayed lower CG methylation compared to WT ES cell at IAP sequences, it suggests that complete CG methylation is not a prerequisite for high nucleosome occupancy. Upon RA treatment, we observed reduced nucleosome occupancy in KO ES cells compared to WT ES at IAP sequences (59% versus 98%) and Line1 elements (59% versus 85%) (Figure 5). These results indicate that nucleosome occupancy is dependent on Lsh at the examined repeat sequences in differentiated ES cells.

**Figure 6.**
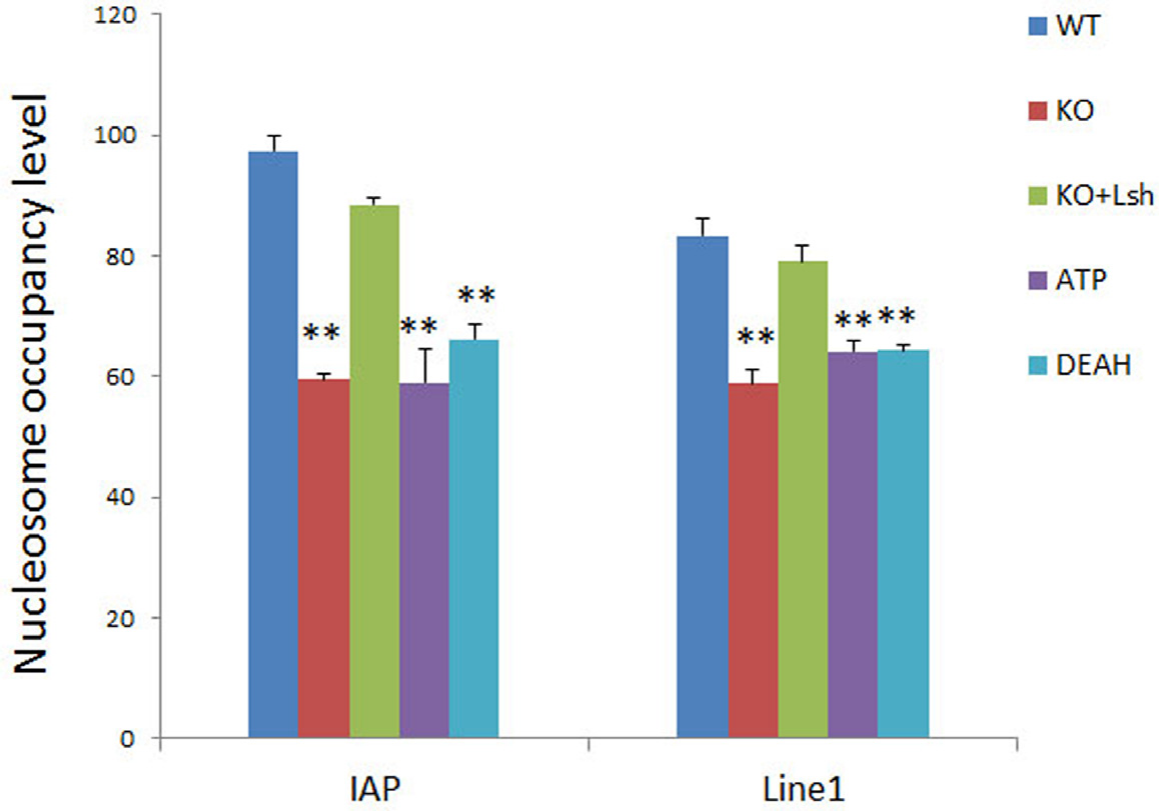
Summary of nucleosome occupancy in dependence of Lsh function. Bar graph representing a summary of NOMe assays at IAP and Line1 sequences as described in Figure 5 for RA-treated ES cells of wild-type origin (WT), KO ES cells (KO) and KO ES cells re-expressing full-length wild-type Lsh (KO+Lsh), the ATP mutant (ATP) or the DEAH domain deletion (DEAH). The data show the mean of three independent experiments (WT, KO, KO+Lsh, ATP) or two independent experiments (DEAH). SD: standard deviation. *P < 0.05; **P < 0.01.

In order to demonstrate that nucleosome occupancy is induced by Lsh, we performed the NOMe assay in differentiated KO ES cells that had been transfected with a full-length wild-type Lsh expression vector. We found that wild-type Lsh was able to restore nucleosome occupancy (from 59 to 87%) at IAP sequences and (from 59 to 79%) at Line1 sequences indicating that nucleosome distribution is dynamic and depends directly on the presence of the Lsh protein (Figure 5A and B). Next, we examined the role of ATP binding site and the DEAH motif of Lsh. The ATP binding site is critical for ATP hydrolysis and both domains are important for chromatin remodeling function of SNF2 proteins. While wild-type Lsh restored nucleosome occupancy effectively in RA-treated KO ES cells, the ATP mutant failed to restore nucleosome occupancy to wild-type levels at IAP (66% versus 87%) and Line1 sequences (65% versus 79%) (Figures 5 and 6). Likewise, the DEAH mutant showed only restoration to 64% (IAP and Line1) far below the nucleosome occupancy obtained with wild-type Lsh (Figures 5 and 6).

These results suggest that nucleosome occupancy at repeat sequences requires a functional ATP site and DEAH domain of the Lsh protein.

Altogether, CG methylation and association of Dnmt3b depend on ATP function of Lsh at repeat sequences in differentiated ES cells. Moreover, ATP function of Lsh is required for altering nucleosome occupancy at repeat elements upon ES differentiation.

## DISCUSSION

Here, we report that the ATP binding site of the Lsh protein is critical to promote DNA methylation at repeat sequences using a dynamic methylation assay in ES cells. We also demonstrate that Lsh is localized at repeat sequences and Lsh influences nucleosome density at those genomic loci which suggests a direct (local) effect of Lsh. We show that Lsh alters nucleosome density in an ATP-dependent manner implying that Lsh plays a chromatin remodeling role at repeat elements. Our data suggest that nucleosome remodeling is the primary molecular function of Lsh which may in turn influence cytosine methylation at repeat elements.

### Lsh and heterochromatin

Lsh has been thought as guardian of heterochromatin (41). Heterochromatin is a compact chromatin structure which tends to be located at the nuclear periphery and impedes the association of the transcriptional machinery with DNA sequences (42). Lsh localizes to major satellite sequences that are embedded in constitutive pericentric heterochromatin and to minor satellite sequences present at centromeric chromatin, and depletion of Lsh leads to transcriptional re-activation of satellite sequences (43). Furthermore, Lsh controls genome wide DNA methylation and silencing of multiple types of repeat sequences, including retroviral-like sequences (16,18). Lsh deficient cells display reduced DNA methylation in a nuclear compartment, which overlaps with lamin B1 attachment regions that comprise repressed chromatin (18). Consistent with the notion that Lsh is associated with repressed chromatin, we report here that Lsh maintained nucleosome density at repeat sequences. The length of internucleosome linker DNA and the regularity of the internucleosome space are thought to influence higher-order folding of nucleosomes and ultimately the compaction of chromatin. Thus Lsh may not only contribute to heterochromatin formation by regulating DNA methylation, but also by modulating nucleosome occupancy.

Several SNF2 factors have been reported to promote heterochromatin formation. *In vitro*, SNF2 homologs are the ATP-driven motors of chromatin that can translocate along DNA and disrupt protein–DNA interactions (1,3). Thus, chromatin remodeling proteins can alter nucleosome positions, compositions and structure. The function *in vivo* is less well understood. Two well-characterized chromatin remodeling complexes have been noted to have opposing effects on chromatin structure (44). The SNF/SWI-like complexes can grant or facilitate access of DNA binding factors to specific DNA sequences playing a role in transcriptional regulation. In contrast, ACF-like complexes may promote higher-order chromatin folding and play a role in heterochromatin formation (44). For example, ACF/CHRAC (45) promotes the assembly of nucleosome arrays *in vitro*. *In vivo*, it is critical for the periodic spacing of nucleosomes and the formation of repressed chromatin (46,47). Mutation of Acf1 (a component of the ACF complex) leads to reduced silencing of sequences embedded in pericentromeric heterochromatin and of polycomb target genes (37,46,48). The SHREC complex contains a chromatin remodeling enzyme subunit called Mit1 which promotes heterochromatin formation. Mit1 function leads to regularly spaced nucleosome arrays, chromatin compaction and transcriptional silencing (40). Thus the removal of nucleosome free regions is a common feature of silencing factors that are involved in heterochromatin formation (49). Likewise, we observed that Lsh induced higher nucleosome occupancy and reduced the appearance of nucleosome free regions.

### DNA methylation and Lsh function

DNA methylation defects are a prominent epigenetic feature in Lsh mutant cells (16,18,25,29). Using a dynamic methylation assay, we demonstrate that establishment of complete CG methylation patterns upon cellular differentiation requires Lsh and its ATP function. ChIPs analysis demonstrated Lsh association at repeat sequences in undifferentiated ES cells (Figure 3). The presence of Lsh at genomic sites that undergo *de novo* methylation suggests a direct (local) involvement of Lsh, as opposed to inducing a factor that in turn promotes DNA methylation.

There are several possibilities to explain how Lsh may promote DNA methylation. A higher nucleosome density may increase the local Dnmt concentration since Dnmts are anchored *in vivo* by nucleosomes and this allows a closer proximity to target sequences (50–52). Dnmts associate with unmodified H3 tails and can perform *de novo* methylation on nucleosome substrates, as has been shown *in vitro* (53,54). Some SNF2 factors can enhance Dnmts affinity to mononucleosomes (54) and SNF2 factors can improve methylation of nucleosome DNA *in vitro* (31). Mit1, an SNF2 factor that reduces nucleosome free regions and increases nucleosome occupancy induces partial unwrapping of nucleosome which may facilitate access of Dnmts to DNA sequences that enter or exit the nucleosome (39). In addition to its chromatin remodeling activity, Lsh may act as a scaffolding protein and directly facilitate the recruitment of Dnmts (28). Finally, Lsh may influence the organization of chromatin into nuclear compartments (18) and may direct the position of Lsh bound repeat sequences to Dnmt-rich regions.

Consistent with the hypothesis that increased Dnmts association may promote cytosine methylation, we detected enhanced Dnmt3b association in the presence of Lsh and reduced recruitment in Lsh deficient cells. However, we do not have direct evidence that DNA methylation of repeat sequences depends on Dnmt3b. A recent publication shows DNA hypomethylation at satellites and IAP sequences in Dnmt3b^−/−^ cells, albeit less severe compared to Lsh^−/−^ cells (16). However, the redundancy of DNA methylation pathways may obscure the role of a single Dnmt, for example, Dnmt3a may, in part, compensate for the loss of Dnmt3b.

### The relationship between DNA methylation and nucleosomes

The link between DNA methylation and chromatin compaction is in part influenced at the nucleosome level. DNA methylation patterns can be instructional in positioning nucleosomes (55,56). Using methylated and unmethylated substrates for nucleosome reconstitution, a subset of nucleosomes was found sensitive to cytosine methylation (55). Methylated CGs positioned at the minor groove, increase the affinity of DNA to histones, thus favoring specific nucleosome positions. In another study, purified genomic DNA (containing native DNA methylation pattern) was assembled into nucleosome arrays, and the same preferential positioning as *in vivo* was detected *in vitro* (57). These results support the notion that DNA methylation influences nucleosome pattering. Furthermore, the rigidity of DNA imposed by CG methylation may disfavor the bending on the nucleosome surface. This can lead to detachment of DNA from the histone core and may destabilize nucleosomes (38,58–60). Nucleosome instability may include repositioning of nucleosomes or rotation of DNA around the histone core. Computational studies suggest lower variability in nucleosome positions for methylated DNA compared to unmethylated DNA (61). The binding of some transcription factors can be directly modulated by DNA methylation (62) and the binding of DNA binding factors may impose repositioning of nucleosomes or create nucleosome-depleted regions (63–65).

On the other hand, several studies suggested that nucleosome occupancy influences DNA methylation patterning *in vivo* and *in vitro* (31,51,66–68). The fact that the bona fide chromatin remodeling factor DDM1 regulates DNA methylation patterning in Arabidopsis thaliana supports this notion. We demonstrate here that the ATP function of Lsh is critical for nucleosome density and for complete cytosine methylation at specific loci. This implies that chromatin remodeling mediated by Lsh is critical for the establishment of DNA methylation patterns. The bilateral relationship between nucleosomes and DNA methylation indicates that chromatin structure depends on a complex of epigenetic modifiers, and all components of this intricate network are critical for cellular differentiation.

DNA methylation is established during cellular differentiation, is unique for each tissue type and represents part of the epigenetic memory. Identifying auxiliary proteins that facilitate DNA methylation, that participate in gene silencing pathways and modify chromatin structure, contributes to our understanding how epigenetic information is created and maintained to grant a stable cellular phenotype and normal embryogenesis.

## SUPPLEMENTARY DATA

Supplementary Data are available at NAR Online.

SUPPLEMENTARY DATA

## References

[1] Clapier C.R., Cairns B.R. (2009). The biology of chromatin remodeling complexes. Annu. Rev. Biochem..

[2] Ho L., Crabtree G.R. (2010). Chromatin remodelling during development. Nature.

[3] Hopfner K.P., Gerhold C.B., Lakomek K., Wollmann P. (2012). Swi2/Snf2 remodelers: hybrid views on hybrid molecular machines. Curr. Opin. Struct. Biol..

[4] Hargreaves D.C., Crabtree G.R. (2011). ATP-dependent chromatin remodeling: genetics, genomics and mechanisms. Cell Res..

[5] Corona D.F., Langst G., Clapier C.R., Bonte E.J., Ferrari S., Tamkun J.W., Becker P.B. (1999). ISWI is an ATP-dependent nucleosome remodeling factor. Mol. Cell.

[6] Mueller-Planitz F., Klinker H., Becker P.B. (2013). Nucleosome sliding mechanisms: new twists in a looped history. Nat. Struct. Mol. Biol..

[7] Dennis K., Fan T., Geiman T., Yan Q., Muegge K. (2001). Lsh, a member of the SNF2 family, is required for genome-wide methylation. Genes Dev..

[8] Eisen J.A., Sweder K.S., Hanawalt P.C. (1995). Evolution of the SNF2 family of proteins: subfamilies with distinct sequences and functions. Nucleic Acids Res..

[9] Jeddeloh J.A., Stokes T.L., Richards E.J. (1999). Maintenance of genomic methylation requires a SWI2/SNF2-like protein. Nat. Genet..

[10] Ehrlich M., Lacey M. (2013). DNA methylation and differentiation: silencing, upregulation and modulation of gene expression. Epigenomics.

[11] Jurkowska R.Z., Jurkowski T.P., Jeltsch A. (2011). Structure and function of mammalian DNA methyltransferases. Chembiochem.

[12] Robertson K.D. (2005). DNA methylation and human disease. Nat. Rev. Genet..

[13] Smith Z.D., Chan M.M., Mikkelsen T.S., Gu H., Gnirke A., Regev A., Meissner A. (2012). A unique regulatory phase of DNA methylation in the early mammalian embryo. Nature.

[14] Lister R., Pelizzola M., Dowen R.H., Hawkins R.D., Hon G., Tonti-Filippini J., Nery J.R., Lee L., Ye Z., Ngo Q.M. (2009). Human DNA methylomes at base resolution show widespread epigenomic differences. Nature.

[15] Lister R., Pelizzola M., Kida Y.S., Hawkins R.D., Nery J.R., Hon G., Antosiewicz-Bourget J., O'Malley R., Castanon R., Klugman S. (2011). Hotspots of aberrant epigenomic reprogramming in human induced pluripotent stem cells. Nature.

[16] Dunican D.S., Cruickshanks H.A., Suzuki M., Semple C.A., Davey T., Arceci R.J., Greally J., Adams I.R., Meehan R.R. (2013). Lsh regulates LTR retrotransposon repression independently of Dnmt3b function. Genome Biol..

[17] Stroud H., Greenberg M.V., Feng S., Bernatavichute Y.V., Jacobsen S.E. (2013). Comprehensive analysis of silencing mutants reveals complex regulation of the Arabidopsis methylome.

[18] Yu W., McIntosh C., Lister R., Zhu I., Han Y., Ren J., Landsman D., Lee E., Briones V., Terashima M. (2014). Genome-wide DNA methylation patterns in LSH mutant reveals de-repression of repeat elements and redundant epigenetic silencing pathways.

[19] Geiman T.M., Tessarollo L., Anver M.R., Kopp J.B., Ward J.M., Muegge K. (2001). Lsh, a SNF2 family member, is required for normal murine development. Biochim. Biophys. Acta.

[20] Sun L.Q., Lee D.W., Zhang Q., Xiao W., Raabe E.H., Meeker A., Miao D., Huso D.L., Arceci R.J. (2004). Growth retardation and premature aging phenotypes in mice with disruption of the SNF2-like gene, PASG. Genes Dev..

[21] De La Fuente R., Baumann C., Fan T., Schmidtmann A., Dobrinski I., Muegge K. (2006). Lsh is required for meiotic chromosome synapsis and retrotransposon silencing in female germ cells. Nat. Cell Biol..

[22] Fan T., Schmidtmann A., Xi S., Briones V., Zhu H., Suh H.C., Gooya J., Keller J.R., Xu H., Roayaei J. (2008). DNA hypomethylation caused by Lsh deletion promotes erythroleukemia development. Epigenetics.

[23] Xi S., Geiman T.M., Briones V., Guang Tao Y., Xu H., Muegge K. (2009). Lsh participates in DNA methylation and silencing of stem cell genes. Stem Cells.

[24] Yu W., Briones V., Lister R., McIntosh C., Han Y., Lee E.Y., Ren J., Terashima M., Leighty R.M., Ecker J.R. (2014). CG hypomethylation in Lsh-/- mouse embryonic fibroblasts is associated with de novo H3K4me1 formation and altered cellular plasticity. Proc. Natl. Acad. Sci. U.S.A..

[25] Tao Y., Xi S., Shan J., Maunakea A., Che A., Briones V., Lee E.Y., Geiman T., Huang J., Stephens R. (2011). Lsh, chromatin remodeling family member, modulates genome-wide cytosine methylation patterns at nonrepeat sequences. Proc. Natl. Acad. Sci. U.S.A..

[26] Yan Q., Huang J., Fan T., Zhu H., Muegge K. (2003). Lsh, a modulator of CpG methylation, is crucial for normal histone methylation. EMBO J..

[27] Xi S., Zhu H., Xu H., Schmidtmann A., Geiman T.M., Muegge K. (2007). Lsh controls Hox gene silencing during development. Proc. Natl. Acad. Sci. U.S.A..

[28] Myant K., Stancheva I. (2008). LSH cooperates with DNA methyltransferases to repress transcription. Mol. Cell. Biol..

[29] Myant K., Termanis A., Sundaram A.Y., Boe T., Li C., Merusi C., Burrage J., de Las Heras J.I., Stancheva I. (2011). LSH and G9a/GLP complex are required for developmentally programmed DNA methylation. Genome Res..

[30] Brzeski J., Jerzmanowski A. (2003). Deficient in DNA methylation 1 (DDM1) defines a novel family of chromatin-remodeling factors. J. Biol. Chem..

[31] Felle M., Hoffmeister H., Rothammer J., Fuchs A., Exler J.H., Langst G. (2011). Nucleosomes protect DNA from DNA methylation in vivo and in vitro. Nucleic Acids Res..

[32] Ying Q.L., Wray J., Nichols J., Batlle-Morera L., Doble B., Woodgett J., Cohen P., Smith A. (2008). The ground state of embryonic stem cell self-renewal. Nature.

[33] Geiman T.M., Muegge K. (2000). Lsh, an SNF2/helicase family member, is required for proliferation of mature T lymphocytes. Proc. Natl. Acad. Sci. U.S.A..

[34] Li L., Sun L., Gao F., Jiang J., Yang Y., Li C., Gu J., Wei Z., Yang A., Lu R. (2010). Stk40 links the pluripotency factor Oct4 to the Erk/MAPK pathway and controls extraembryonic endoderm differentiation. Proc. Natl. Acad. Sci. U.S.A..

[35] Taberlay P.C., Kelly T.K., Liu C.C., You J.S., De Carvalho D.D., Miranda T.B., Zhou X.J., Liang G., Jones P.A. (2011). Polycomb-repressed genes have permissive enhancers that initiate reprogramming. Cell.

[36] Kelly T.K., Liu Y., Lay F.D., Liang G., Berman B.P., Jones P.A. (2012). Genome-wide mapping of nucleosome positioning and DNA methylation within individual DNA molecules. Genome Res..

[37] Kelly T.K., Miranda T.B., Liang G., Berman B.P., Lin J.C., Tanay A., Jones P.A. (2010). H2A.Z maintenance during mitosis reveals nucleosome shifting on mitotically silenced genes. Mol. Cell.

[38] Smit A.F. (1999). Interspersed repeats and other mementos of transposable elements in mammalian genomes. Curr. Opin. Genet. Dev..

[39] Creamer K.M., Job G., Shanker S., Neale G.A., Lin Y.C., Bartholomew B., Partridge J.F. (2014). The Mi-2 homolog Mit1 actively positions nucleosomes within heterochromatin to suppress transcription. Mol. Cell. Biol..

[40] Sugiyama T., Cam H.P., Sugiyama R., Noma K., Zofall M., Kobayashi R., Grewal S.I. (2007). SHREC, an effector complex for heterochromatic transcriptional silencing. Cell.

[41] Muegge K. (2005). Lsh, a guardian of heterochromatin at repeat elements. Biochem. Cell Biol..

[42] Woodcock C.L., Ghosh R.P. (2010). Chromatin higher-order structure and dynamics. Cold Spring Harb. Perspect. Biol..

[43] Huang J., Fan T., Yan Q., Zhu H., Fox S., Issaq H.J., Best L., Gangi L., Munroe D., Muegge K. (2004). Lsh, an epigenetic guardian of repetitive elements. Nucleic Acids Res..

[44] Narlikar G.J., Sundaramoorthy R., Owen-Hughes T. (2013). Mechanisms and functions of ATP-dependent chromatin-remodeling enzymes. Cell.

[45] Ito T., Levenstein M.E., Fyodorov D.V., Kutach A.K., Kobayashi R., Kadonaga J.T. (1999). ACF consists of two subunits, Acf1 and ISWI, that function cooperatively in the ATP-dependent catalysis of chromatin assembly. Genes Dev..

[46] Fyodorov D.V., Blower M.D., Karpen G.H., Kadonaga J.T. (2004). Acf1 confers unique activities to ACF/CHRAC and promotes the formation rather than disruption of chromatin in vivo. Genes Dev..

[47] Varga-Weisz P.D., Wilm M., Bonte E., Dumas K., Mann M., Becker P.B. (1997). Chromatin-remodelling factor CHRAC contains the ATPases ISWI and topoisomerase II. Nature.

[48] Liu Y.I., Chang M.V., Li H.E., Barolo S., Chang J.L., Blauwkamp T.A., Cadigan K.M. (2008). The chromatin remodelers ISWI and ACF1 directly repress Wingless transcriptional targets. Dev. Biol..

[49] Garcia J.F., Dumesic P.A., Hartley P.D., El-Samad H., Madhani H.D. (2010). Combinatorial, site-specific requirement for heterochromatic silencing factors in the elimination of nucleosome-free regions. Genes Dev..

[50] Chen T., Ueda Y., Xie S., Li E. (2002). A novel Dnmt3a isoform produced from an alternative promoter localizes to euchromatin and its expression correlates with active de novo methylation. J. Biol. Chem..

[51] Jeong S., Liang G., Sharma S., Lin J.C., Choi S.H., Han H., Yoo C.B., Egger G., Yang A.S., Jones P.A. (2009). Selective anchoring of DNA methyltransferases 3A and 3B to nucleosomes containing methylated DNA. Mol. Cell. Biol..

[52] Sharma S., De Carvalho D.D., Jeong S., Jones P.A., Liang G. (2011). Nucleosomes containing methylated DNA stabilize DNA methyltransferases 3A/3B and ensure faithful epigenetic inheritance. PLoS Genet..

[53] Gowher H., Stockdale C.J., Goyal R., Ferreira H., Owen-Hughes T., Jeltsch A. (2005). De novo methylation of nucleosomal DNA by the mammalian Dnmt1 and Dnmt3A DNA methyltransferases. Biochemistry.

[54] Robertson A.K., Geiman T.M., Sankpal U.T., Hager G.L., Robertson K.D. (2004). Effects of chromatin structure on the enzymatic and DNA binding functions of DNA methyltransferases DNMT1 and Dnmt3a in vitro. Biochem. Biophys. Res. Commun..

[55] Collings C.K., Waddell P.J., Anderson J.N. (2013). Effects of DNA methylation on nucleosome stability. Nucleic Acids Res..

[56] Portela A., Liz J., Nogales V., Setien F., Villanueva A., Esteller M. (2013). DNA methylation determines nucleosome occupancy in the 5’-CpG islands of tumor suppressor genes. Oncogene.

[57] Huff J.T., Zilberman D. (2014). Dnmt1-independent CG methylation contributes to nucleosome positioning in diverse eukaryotes. Cell.

[58] Jimenez-Useche I., Ke J., Tian Y., Shim D., Howell S.C., Qiu X., Yuan C. (2013). DNA methylation regulated nucleosome dynamics. Sci. Rep..

[59] Perez A., Castellazzi C.L., Battistini F., Collinet K., Flores O., Deniz O., Ruiz M.L., Torrents D., Eritja R., Soler-Lopez M. (2012). Impact of methylation on the physical properties of DNA. Biophys. J..

[60] Portella G., Battistini F., Orozco M. (2013). Understanding the connection between epigenetic DNA methylation and nucleosome positioning from computer simulations. PLoS Comput. Biol..

[61] Minary P., Levitt M. (2014). Training-free atomistic prediction of nucleosome occupancy. Proc. Natl. Acad. Sci. U.S.A..

[62] Mann I.K., Chatterjee R., Zhao J., He X., Weirauch M.T., Hughes T.R., Vinson C. (2013). CG methylated microarrays identify a novel methylated sequence bound by the CEBPB|ATF4 heterodimer that is active in vivo. Genome Res..

[63] Kaplan N., Moore I.K., Fondufe-Mittendorf Y., Gossett A.J., Tillo D., Field Y., LeProust E.M., Hughes T.R., Lieb J.D., Widom J. (2009). The DNA-encoded nucleosome organization of a eukaryotic genome. Nature.

[64] Lee W., Tillo D., Bray N., Morse R.H., Davis R.W., Hughes T.R., Nislow C. (2007). A high-resolution atlas of nucleosome occupancy in yeast. Nat. Genet..

[65] Zaret K.S., Carroll J.S. (2011). Pioneer transcription factors: establishing competence for gene expression. Genes Dev..

[66] Chodavarapu R.K., Feng S., Bernatavichute Y.V., Chen P.Y., Stroud H., Yu Y., Hetzel J.A., Kuo F., Kim J., Cokus S.J. (2010). Relationship between nucleosome positioning and DNA methylation. Nature.

[67] Jiang Y., Schneck J.L., Grimes M., Taylor A.N., Hou W., Thrall S.H., Sweitzer S.M. (2011). Methyltransferases prefer monomer over core-trimmed nucleosomes as in vitro substrates. Anal. Biochem..

[68] Jin B., Li Y., Robertson K.D. (2011). DNA methylation: superior or subordinate in the epigenetic hierarchy. Genes Cancer.

