# Supplementary material for: The ATP binding site of the chromatin remodeling homolog Lsh is required for nucleosome density and de novo DNA methylation at repeat sequences
Source: Nucleic Acids Res. 2015 Jan 10;43(3):1444–55. doi: 10.1093/nar/gku1371 (PMC4330352; doi:10.1093/nar/gku1371)
Supplement: SUPPLEMENTARY DATA [file supp_43_3_1444__index.html]

The ATP binding site of the chromatin remodeling homolog Lsh is required for nucleosome density and de novo DNA methylation at repeat sequences — The ATP binding site of the chromatin remodeling homolog Lsh is required for nucleosome density and de novo DNA methylation at repeat sequences — SUPPLEMENTARY DATA 

# The ATP binding site of the chromatin remodeling homolog Lsh is required for nucleosome density and *de novo* DNA methylation at repeat sequences

## SUPPLEMENTARY DATA

**Files in this Data Supplement:**

- SUPPLEMENTARY DATA
