# Supplementary material for: The ATP binding site of the chromatin remodeling homolog Lsh is required for nucleosome density and de novo DNA methylation at repeat sequences
Source: Nucleic Acids Res. 2015 Jan 10;43(3):1444–55. doi: 10.1093/nar/gku1371 (PMC4330352; doi:10.1093/nar/gku1371)
Supplement: SUPPLEMENTARY DATA [file supp_gku1371_nar-03020-m-2014-File008.pdf]

## Supplemental Figure Legends

### Supplemental Fig 1: Generation of Lsh-/- (KO) ES cells and Lsh+/+ (WT) ES cells from day3.5 blastocysts and characterization of markers for pluripotency

A. Real-time PCR analysis for detection of Lsh mRNA in two independently derived undifferentiated KO ES cells and WT ES cells.

B. Western analysis for detection of Lsh protein in two independently derived KO ES cells and WT ES cells.

C. Cellular morphology of KO ES and WT ES cells.

D. Alkaline phosphatase staining of KO ES and WT ES cells.

E. Immunefluorescence staining for detection of pluripotency markers Sox2, Nanog, Oct4 and SSEA1 in undifferentiated KO ES and WT ES cells.

F. Bisulfite sequencing analysis of the Oct4 promoter region in undifferentiated WT and KO ES cells.

### Supplemental Fig 2: Differentiation of Lsh-/- (KO) ES cells and Lsh+/+ (WT) ES cells

A. Growth curve of WT and KO ES cells and KO ES cells re-expressing Lsh (KO+Lsh).

B. Protocol for differentiation of ES cells to the neuroepithelial lineage with embryoid body formation followed by RA treatment

C. Detection of neuronal marker expression Tubb3 and Nestin in differentiated KO ES cells and WT ES cells. Nuclei were stained with DAPI.

D. Real-time PCR analysis for quantification of several neuronal lineage genes in differentiated WT and KO ES cells, including Neurexin 1 (Nrxn1), Neuron cell adhesion molecule (Nrcam),

and Reelin (Reln). RNA was normalized to Gapdh. SD (standard deviation) is shown from two independent experiments.

**Supplemental Fig 3 : CG methylation at repeat sequences in undifferentiated ES cell.**

A. Bisulfite sequencing analysis of IAP, minor satellite sequences and Line1 elements in undifferentiated WT ES cells (WT), KO ES cells (KO) and KO ES cells re-expressing full length Lsh protein (KO+Lsh). The black circles represent CG methylation, while the white circles indicate no methylation at specific CpG sites. Bisulfite sequencing was not examined for Line1 in KO+ES cells, since WT and KO ES showed undistinguishable DNA methylation in undifferentiated cells.

B. Bar graph of CG methylation level in undifferentiated ES cells compared to differentiated ES cells with WT ES cells (WT), KO ES cells (KO) and KO ES cells re-expressing full length wild type Lsh protein (KO+Lsh) at IAP sequences, minor satellite sequences and Line1 elements. The bar graphs represents the mean of three independent experiments (WT and KO for Line1and IAP) or two independent experiments (minor satellite and KO+Lsh), with each including at least ten sequenced clones. For better comparison, the data from Figure 1E has been added. SD, standard deviation. $^*p<0.05$; $^{**}p<0.01$.

**Supplemental Fig 4: Nucleosome occupancy at IAP and Line1 elements in dependence of Lsh.**

A. NOMe assay for IAP (left panel) and Line 1 (right panel) elements in undifferentiated WT ES cells (WT) and KO ES cells (KO). The GpC methylation profiles are shown on the left hand and represents GpC sites that are not methylated (white circle) or methylated (green-filled circles) indicating the accessibility of those GpC sites to GpC methyltransferase. The areas of inaccessibility, large enough to accommodate a nucleosome are identified as described (35-37) and are covered by pink color. The CpG methylation profiles are shown on the right with methylated (black circle) and unmethylated (white circles) cytosines. Data represent one out of two independent experiments.

B. The bar graph represents the mean of the nucleosome occupancy assay from two independent experiments (described in supplemental Fig4A) with each including at least ten sequenced clones. SD, standard deviation. *$p<0.05$; **$p<0.01$.

Supplement Fig 1

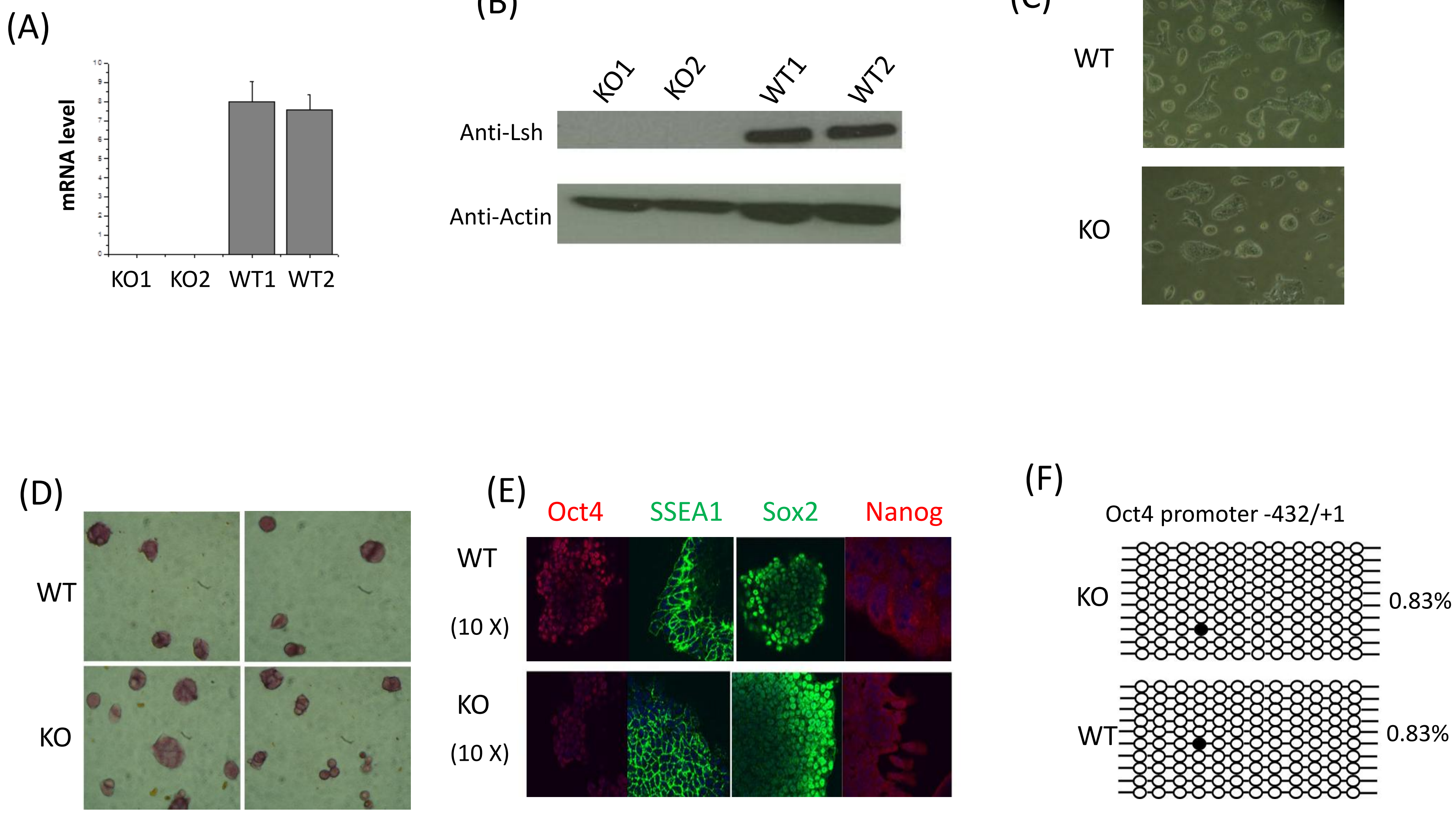

Supplement Fig 2

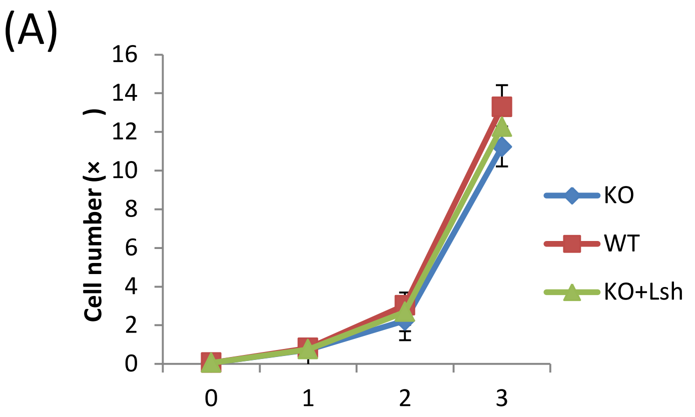


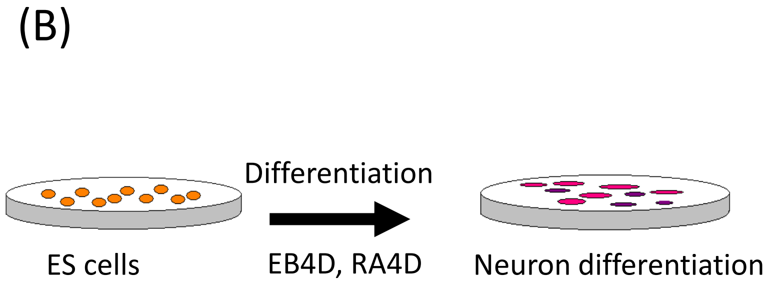


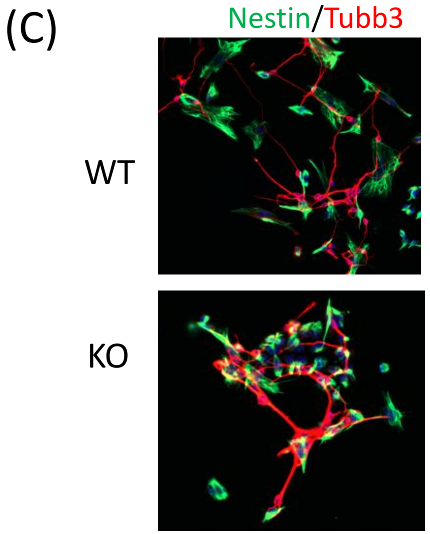


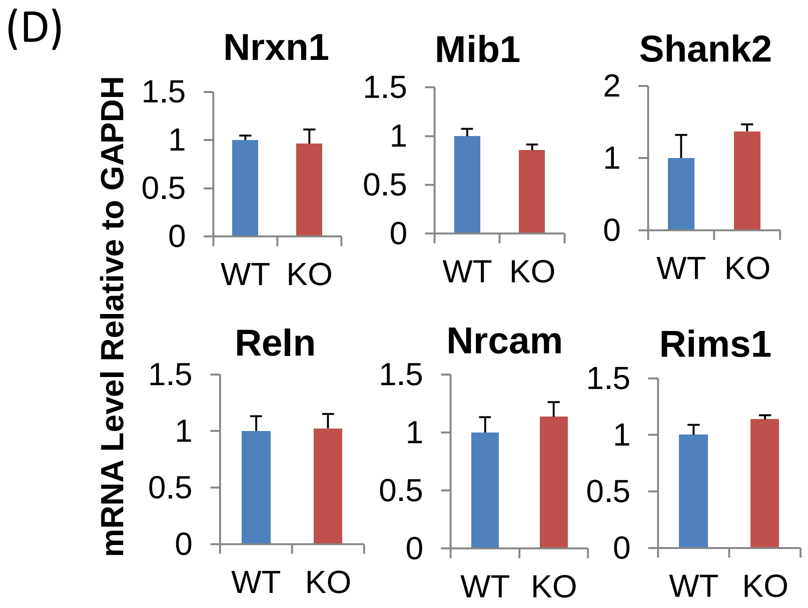

Supplement Fig 3

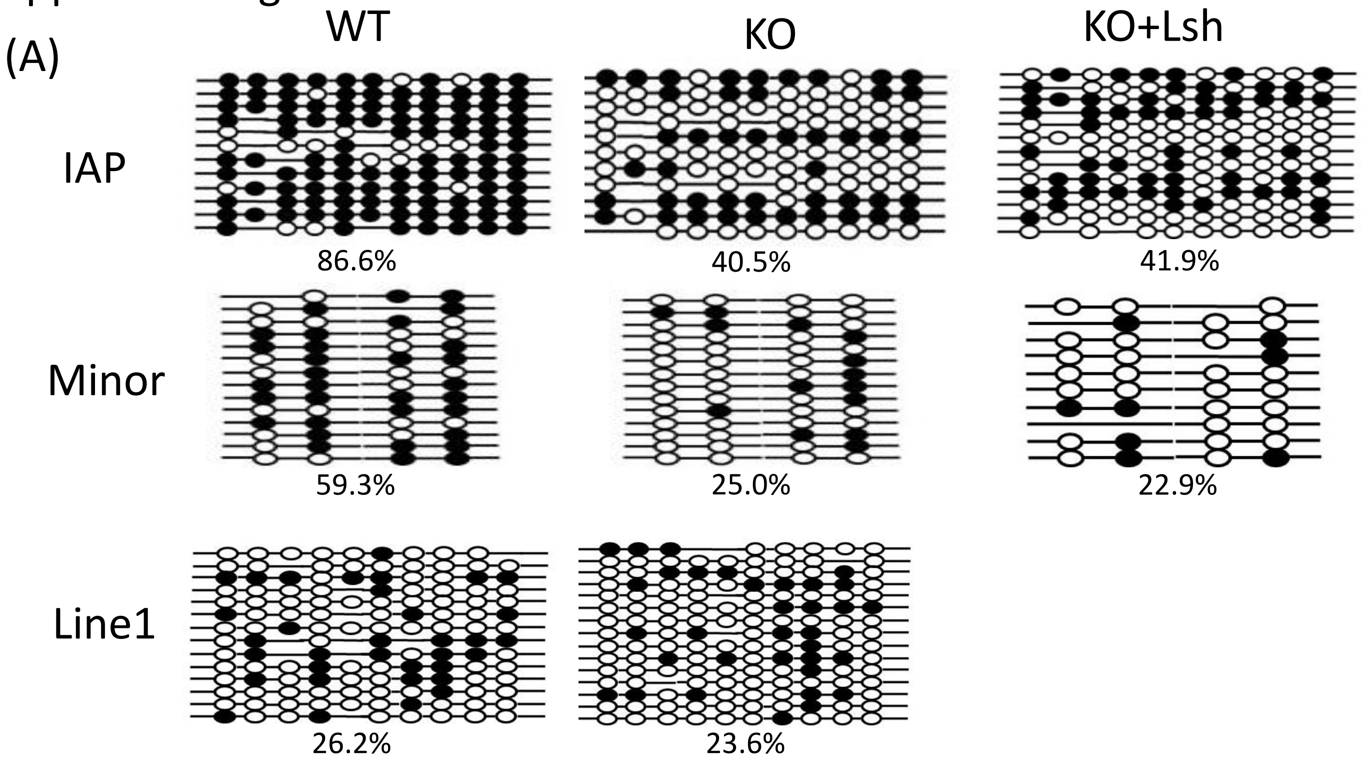


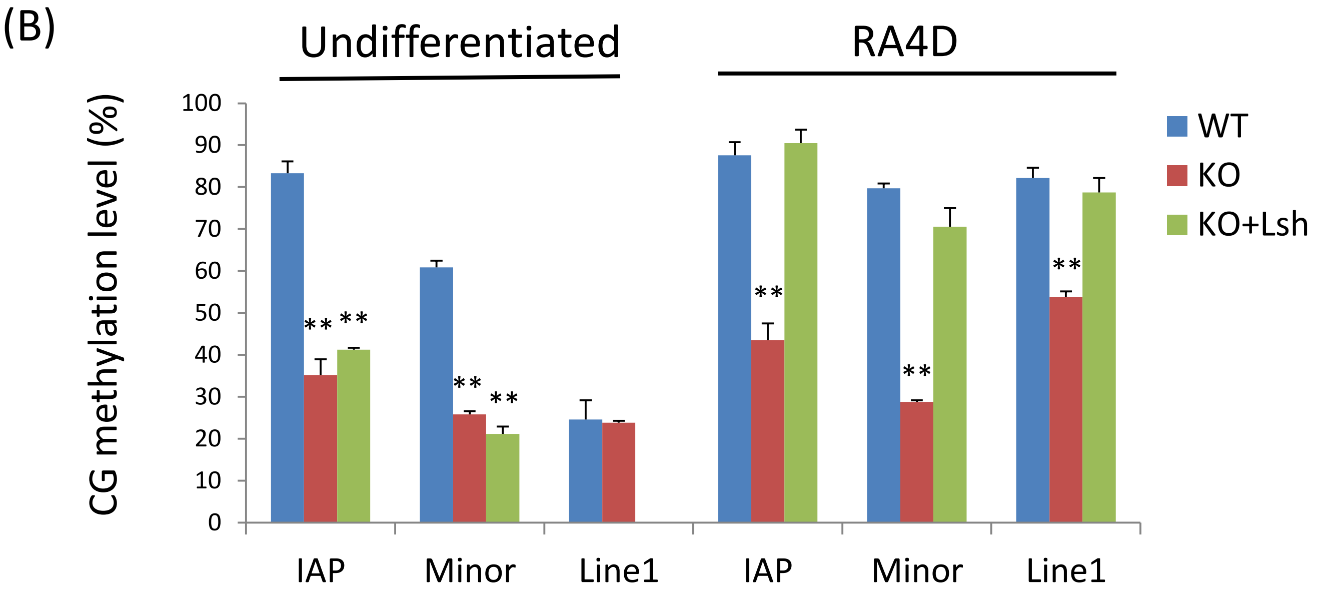

Supplement Fig 4

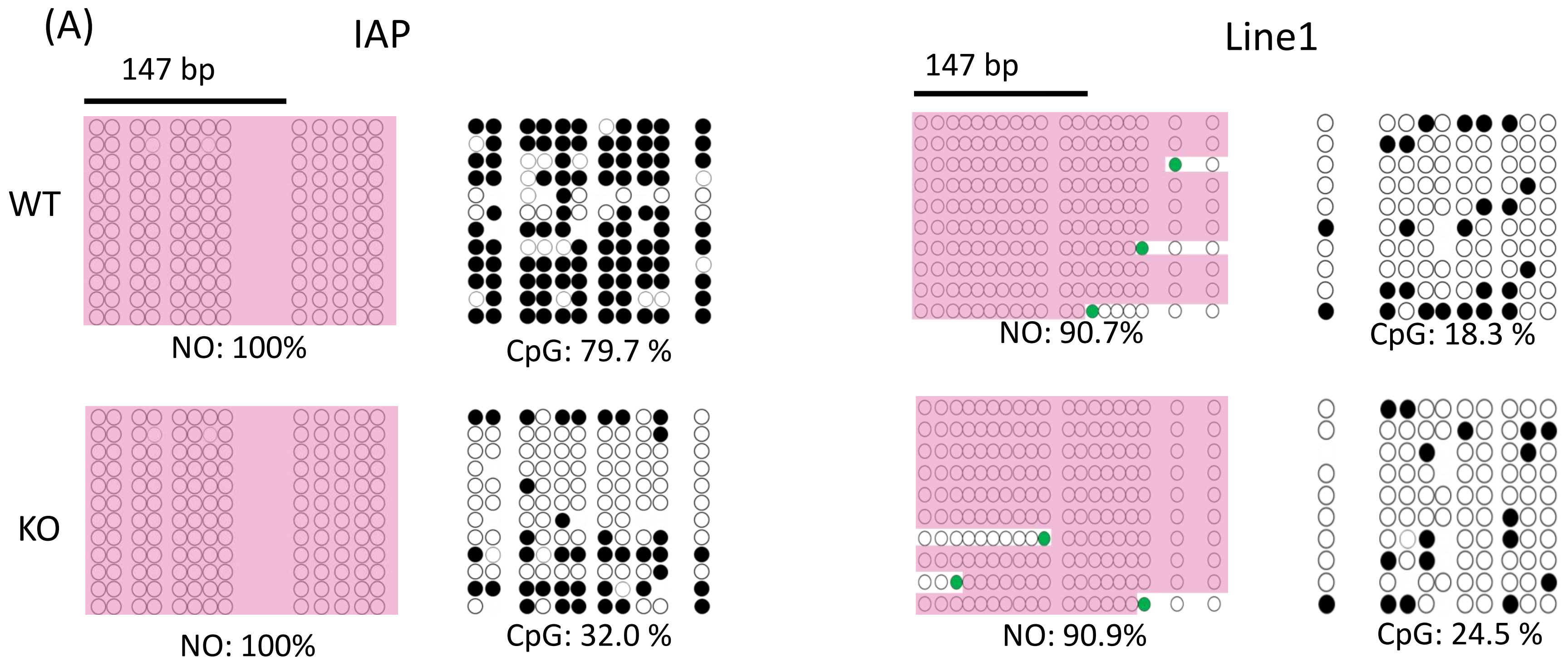


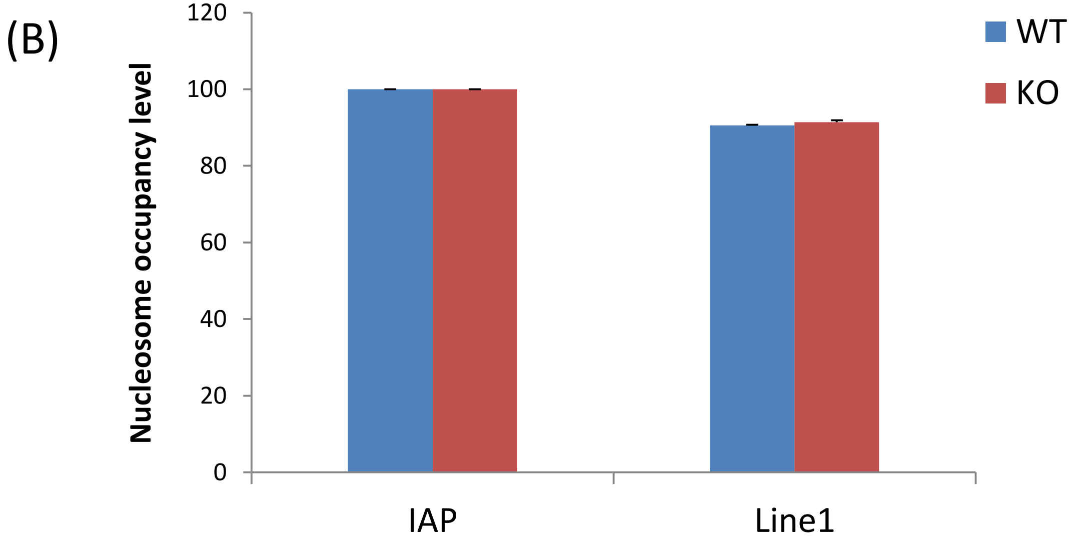

Supplement Table 1

| Name | Bisulfite primer sequences(5'-3') | position |
|---|---|---|
| IAP | F: TTGTGTTTTAAGTGGTAAATAAATAATTTG<br>R: CAAAAAAAACACACAAACCAAAAT | 48-310 |
| Minor | F: GAGAAATATGGAAAATGATAAAAA<br>R: CATTAATATACACTATTCTACAAATCC | 40-271 |
| Line1 | F: TAGGAAATTAGTTTGAATAGGTGAGAGG<br>R: TCAAACACTATATTACTTTAACAATTCCCA | 875-1156 |
| Oct4 | F: ATGGGTTGAAATATTGGGTTTATTTA<br>R: CCACCCTCTAACCTTAACCTCTAAC | -432-1 |
| | | |
| **Name** | **Chip primer sequences(5'-3')** | **position** |
| IAP | F: CAAATAATCTGCGCATATGCCGA<br>R: GACCAGAATCTTCTGCGGCAA | 67-294 |
| Minor | F: TTGTACAACAGTGTATATCAATG<br>R: GTTTCCAACGAATGTGTTTTTCAG | 8-122 |
| Line1 | F: TTCTTCTTCGGCCAGGAGGAGGTC<br>R: GCTGGGTTCTGGTGATGGTGAGTG | 1177-1450 |
| | | |
| **Name** | **Gene expression primer sequences(5'-3')** | |
| Nrxn1 | F: CCAGTGACGATGAGGACATTG<br>R: ATAGAGGAGGATGAGGATGCAC | |
| Mib1 | F: GGTGACCTCAATGAAGAGCTG<br>R: CCAGCACACTGTCCGTTTAC | |
| Shank2 | F: GAGCTAAACTCCATTCTGCAGC<br>R: ACAGTGAAGGTGACCGTCGT | |
| Reln | F: ACTGGAATTGAACCCCAACA<br>R: GCTCACAGAAGGTGACAGCA | |
| Nrcam | F: GAAAAGGAGGATGCTCATGC<br>R: TTCACCCCCTCTCCATAGTC | |
| Rims1 | F: CAAGGCCTATTGGTGACATCC<br>R: CCCACCTTATCATACCACAGC | |
| Lsh | F: GCAGATGAAATGGGTTTGGGA<br>R: GGGTTCCATGATACAGCAGAG | |
